# D-Serine disrupts Cbln1 and GluD1 interaction and affects Cbln1-dependent synaptic effects and nocifensive responses in the central amygdala

**DOI:** 10.1007/s00018-024-05554-z

**Published:** 2025-01-31

**Authors:** Siddhesh S. Sabnis, Kishore Kumar S. Narasimhan, Poojashree B. Chettiar, Gajanan P. Shelkar, Shashank M. Dravid

**Affiliations:** Department of Psychiatry and Behavioral Sciences, Health Sciences Center, School of Medicine, Texas A&M University, 206, Olsen Blvd, Reynolds Medical Sciences Bldg, College Station, TX 77845 USA

**Keywords:** Cbln1, Central amygdala, D-serine, GluD1, Chronic pain, Nocifensive behavior

## Abstract

Ionotropic glutamate receptors (iGluRs) mediate fast excitatory neurotransmission in the nervous system. In addition to NMDA receptor co-agonists, D-serine is a ligand for glutamate delta receptors (GluDs) and interacts with the ligand-binding domain with low affinity. However, D-serine binding does not lead to typical ion channel currents in GluD1 or GluD2 but may contribute to synaptic plasticity. In the developing brain, D-serine binding to GluD2 facilitates long-term depression at parallel fiber-Purkinje cell synapses. However, the influence of D-serine on GluD1's interaction with its amino terminal domain synaptogenic ligand Cbln1 and its subsequent impact on synaptic function and behavior remains unexplored. Here, we found that D-serine inhibited the interaction between Cbln1 and GluD1 in an in vitro cell-binding assay. This effect was concentration-dependent, with an IC_50_ value of ~ 300 µM. Furthermore, in ex vivo central amygdala (CeA) slices application of recombinant Cbln1 (rCbln1), consistent with its synaptogenic property, produced a robust increase in excitatory neurotransmission and GluD1 expression. This effect of rCbln1 was partially blocked by pre-treatment with D-serine. Finally, in behavioral experiments, we observed that the pro-nociceptive effect of intra-CeA injection of rCbln1 was inhibited by pre-treatment with D-serine. In addition, the antinociceptive effect of intra-CeA rCbln1 injection in an inflammatory pain model was blocked by D-serine. Overall, these results demonstrated that D-serine binding to GluD1 reduces its interaction with Cbln1, which may be relevant to synaptic plasticity and behavior.

**Graphical Abstract:**

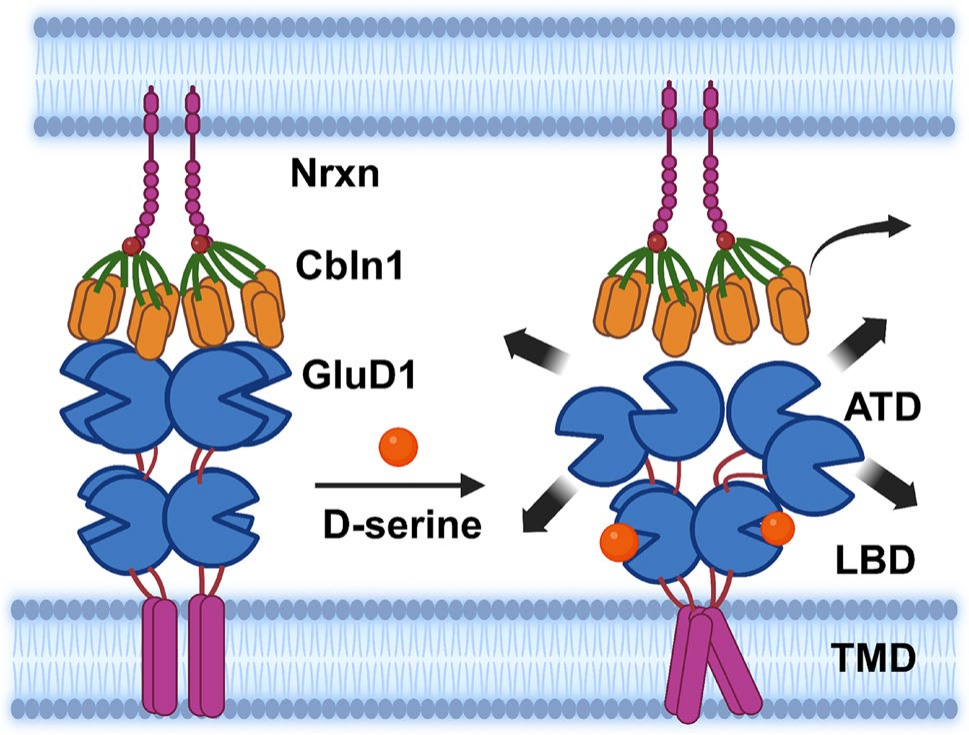

**Supplementary Information:**

The online version contains supplementary material available at 10.1007/s00018-024-05554-z.

## Introduction

Ionotropic glutamate receptors (iGluRs) are ligand-gated ion channels responsible for the fast excitatory synaptic neurotransmission in the central nervous system. The iGluRs are classified into four subfamilies: α-amino-3-hydroxy-5-methyl-4-isoxazolepropionate (AMPA), kainate, N-methyl-D-aspartate (NMDA), and delta receptors. Structurally, iGluRs consist of an extracellular amino terminal domain (ATD), ligand-binding domain (LBD), transmembrane domain (TMD), and intracellular C-terminal domain (CTD) [1]. Although GluDs share ~ 20–30% structural similarity with other iGluRs, they do not function as typical ligand-gated ion channels in a heterologous expression system. Instead, studies have shown that GluDs function as synapse organizers by forming a tripartite bridge by binding to the transsynaptic molecule (Cblns) which in turn bind presynaptic neurexins (Nrxns). This GluD-Cbln-Nrxn transsynaptic network plays a critical role in the formation and maintenance of parallel fiber-Purkinje cells (PF-PC) and thalamostriatal, hippocampal, and parabrachioamygdala synapses [2–7].

Although GluDs do not function as typical ion channels, their LBD is capable of binding endogenous ligands such as D-serine, glycine, and GABA [8, 9]. The binding affinity of D-serine for GluD2, estimated using isothermal titration calorimetry, is ~ 800 µM [8] but is significantly higher for GluD1 (K_d_ = 160 μM) [10]. D-serine induces conformational changes in the receptor, as evidenced by its effect on constitutive currents from mutant GluD2 and GluD1 [8–12]. In the native system, D-serine binding to GluDs has been found to induce currents indirectly by activating metabotropic receptors [13–15]. More recently, it has been suggested that GluDs may function as ligand-gated ion channels when they are in their transsynaptic configuration [16]; however, this finding requires further validation in native systems. Furthermore, the binding of D-serine to GluDs also contributes to synaptic plasticity. For instance, D-serine binding to GluD2 induces long-term depression at the PF-PC synapses in the developing cerebellum [17], and D-serine binding to GluD1 is necessary for the formation of inhibitory synapses [18]. More recently, it has been found that GABA binds to GluD1 with a lower affinity and is important for plasticity at inhibitory synapses [9]. Despite these converging findings on the role of D-serine on GluD function and signaling, it is not well understood whether there is an allosteric interaction between D-serine and Cbln1 binding to GluD1. Such an interaction may affect the synaptogenic activity of Cbln1 and the effect of ligand binding on plasticity. Here, we studied the effect of D-serine on GluD1 interaction with Cbln1 and its downstream effects on synaptic function and behavior. We found that D-serine reduced Cbln1 binding to GluD1 in an in vitro cell-binding assay. Furthermore, D-serine inhibited the increase in excitatory neurotransmission induced by recombinant Cbln1 (rCbln1) in CeA neurons. In addition, D-serine reduced the rCbln1-induced upregulation of GluD1 in the synaptic compartment. Furthermore, we found that D-serine inhibited the pro-nociceptive effect of intra-CeA rCbln1 in naïve animals as well as the antinociceptive effect of rCbln1 in an inflammatory pain model. Together, these results identified a previously unknown interaction between the GluD1 LBD and ATD ligands. We hypothesize that such interactions may play a role in synaptic plasticity in physiological and pathological conditions.

## Methods

### Cell culture

COS-7 cells (ATCC Cat# CRL-1651, RRID: CVCL_0224) were cultured in DMEM supplemented with 10% FBS, 2 mM L-glutamine, and 1% antibiotics in a humidified incubator under 5% CO_2_ at 37 °C.

### Cell binding assay

The interaction between Cbln1-HA (R&D Systems, Minneapolis, MN, USA) and GluD1 was assessed using an in vitro binding assay as previously described with minor modifications [19]. COS-7 cells were cultured on poly l-lysine-coated coverslips and transfected with the rat pCAGGS GluD1 plasmid (gift from Dr. Michisuke Yuzaki) or mutant pCAGGS GluD1R526K using PEI at a ratio of 1:1.5. Following a 48-h transfection period and removal of the transfection reagent, cells were treated with different D-serine concentrations for 30 min followed by recombinant Cbln1 protein (1 μg/ml) in the presence of D-serine (or vehicle) for 4 h. Subsequently, the medium was aspirated, and the cells were rinsed twice with phosphate-buffered saline (PBS, pH 7.4) before fixation with 4% paraformaldehyde (PFA) for 15 min. After fixation, the cells were washed with PBS and incubated with 3% bovine serum albumin (BSA) for 1 h at room temperature to block nonspecific binding sites. Following blocking, the cells were treated with an anti-HA primary antibody (Cat# 2367S, Cell Signaling Technologies, Danvers, MA, USA) for 2 h at room temperature, followed by three PBS washes. Subsequently, the cells were incubated with an anti-mouse secondary antibody conjugated to Alexa Fluor488 (Cat# A21202, Thermo Fisher Scientific, Waltham, MA, USA) for 1 h, followed by three PBS washes. Finally, the coverslips were mounted on glass slides using Fluoromount-G (0100-01, SouthernBiotech, Birmingham, AL, USA), and fluorescent images were captured using a Nikon Eclipse upright microscope.

### Cell binding followed by immunoblotting

Quantitative cell binding was performed with some modifications as described previously [20]. Briefly, HEK293T cells were transfected with pCAGGS GluD1. Following a 48-h transfection period and removal of the transfection reagent, cells were treated with D-serine (or vehicle) for 30 min followed by recombinant Cbln1 protein (1 μg/ml) in the presence of D-serine (or vehicle) for 4 h. Subsequently, the medium was aspirated, and the cells were rinsed thrice with PBS. The cell lysate was collected and subjected to SDS-PAGE. Membranes were probed using 1:1000; mouse monoclonal anti-his-tag (#D-291-3, MBL International Corporation) and 1:1000; guinea pig monoclonal anti-GluD1 (#MSFR102510, Frontiers Institute Co. Ltd.) antibodies.

### Experimental animals

C57BL/6 and GluD1 KO male and female mice were used in this study. All studies were approved by Creighton University and Texas A&M IACUC. All efforts were made to minimize distress and discomfort in the animals. Mice were housed at 22 ± 1 °C with a 12 h light–dark cycle, with access to food and water ad libitum. Mice weighing 20–30 g–2–4 months old were used for all experiments.

### Brain slice electrophysiology

Whole-cell brain slice electrophysiology was conducted to record mEPSC currents from CeA neurons, as previously described [5], with slight modifications. Briefly, mice were anesthetized with isoflurane, followed by decapitation and removal of the brain. The isolated brain was transferred to the ice-cold artificial cerebrospinal fluid (aCSF) of the composition 130 mM NaCl, 24 mM NaHCO_3_, 3.5 mM KCl, 1.25 mM NaH_2_PO_4_, 2.4 mM CaCl_2_, 2.5 mM MgCl_2_ and 10 mM glucose saturated with 95% O_2_/5% CO_2_. 300 μm thick sections were cut using a vibratome (Leica VT1200S; Buffalo Grove, IL, USA). These sections were incubated with either D-serine (300 μM) (or vehicle) for 15 min, followed by the addition of rCbln1 (1 μg/ml) or vehicle and incubation for 30 min. Effect of reversing the drug exposure sequence was tested by incubation with rCbln1 followed by D-serine. Incubation was performed in the presence of MK-801 (50 μM) to block NMDA receptor-mediated activity. Whole-cell patch clamp recordings were obtained from neurons in the lateral capsular region of the CeA (CeLC) in a voltage-clamp configuration with an Axopatch 200 B (Molecular Devices, Sunnyvale, CA, USA). Glass pipette (resistance of 4–6 MΩ) filled with an internal solution consisting of 110 mM Cs gluconate, 30 mM CsCl, 5 mM HEPES, 4 mM NaCl, 0.5 mM CaCl_2_, 2 mM MgCl_2_, 5 mM BAPTA, 2 mM Na_2_ATP, and 0.3 mM Na_2_GTP (pH 7.35) was used for patching. QX314 was added to the internal solution to block voltage-gated sodium channels. The aCSF solution contained 1.5 mM of CaCl_2_ and 1.5 mM of MgCl_2_. mEPSC’s were recorded at holding potential −70 mV in the presence of 0.5 μM tetrodotoxin and 100 μM picrotoxin. The signal was filtered at 5 kHz and digitized at 10 kHz using an Axon Digidata 1440A analog-to-digital board (Molecular Devices). mEPSC recordings were analyzed with MiniAnalysis software (Synaposoft, Atlanta, GA, USA) using a 5-pA amplitude threshold. The frequencies and amplitudes of the miniature currents were measured.

### Immunohistochemistry

Immunohistochemical analysis of GluD1 and PKCδ expression was performed as previously described [5], with minor modifications. Mice were anesthetized with isoflurane and decapitated, their brains were removed, and brain slices (150 μm) were prepared using a vibratome (Leica VT1200S, Buffalo Grove, IL, USA). Brain slices were incubated with D-serine (300 μM) (or vehicle) for 15 min, and then Cbln1 (1 μg/ml) (or vehicle) was added, and slices were incubated for an additional 30 min. These slices were then transferred to a 4% paraformaldehyde solution for 15 min and subjected to immunohistochemistry. After washing with 0.1 M phosphate buffer, sections were incubated in blocking solution containing 10% normal goat serum (Jackson Immuno Research Laboratories Inc., West Grove, PA, Cat # 005-000-121) for 1 h at room temperature. After blocking, the sections were incubated in a cocktail of guinea pig anti-GluD1 (1:500, GluD1C-GP-Af860, Frontier Institute Co. Ltd., Sapporo, Japan), and mouse PKCδ (1:500, Cat # 10,397, BD Biosciences, San Jose, CA, USA) antibodies overnight at 4 °C. The following day, sections were washed and incubated in anti-guinea pig conjugated to Alexa Fluor 488 (1:500, A-11073, Life Technologies) or in a cocktail of goat anti-guinea pig conjugated to Alexa Fluor 488 (1:500, A-11073, Life Technologies) and goat anti-mouse conjugated to Alexa Fluor 594 (1:500, A-11032, Life Technologies) secondary antibodies for 2 h at room temperature. Sections were washed and mounted using Fluoromount-G (Southern Biotech, Birmingham, AL, USA). Confocal images were acquired using a Nikon Eclipse Ti2-E inverted confocal microscope equipped with an AX point scanner. Images of an equivalent region, 1024 × 1024 pixels, were captured using a 60 × oil immersion or 20 × objective at a 1 × zoom. CeA sections were scanned at 0.3 µm intervals along the z-axis, and an optical Sect. (18 µm thick) was taken from each tissue section. GluD1 puncta number and volume were analyzed using NIS-Element image analysis software (Nikon, NY, USA). Images from four mice per group were analyzed and plotted. Images were analyzed by a trained observer who was blinded to the treatments.

### Synaptoneurosomes preparation and immunoblotting

Brain slices were prepared and incubated with drugs as described for the electrophysiology experiments. The CeA was punched with reference to the Allen Brain Atlas and processed for synaptoneurosomes. Synaptoneurosome preparation was performed as previously described [19] with slight modifications. RCeA was homogenized in a synaptoneurosome buffer with following composition: 10 mM HEPES, 1-mM EDTA, 2-mM EGTA, 0.5-mM DTT, 0.5-mM PMSF, 50 μg/ml soybean trypsin inhibitor, 0.25% of phosphatase inhibitor cocktail 2% and 3%, and 0.25% of protease inhibitor cocktail followed by 3 times sonication (FB50, Fisher Scientific, Pittsburgh, PA, USA). Samples obtained from the previous step were filtered twice using three layers of a pre-wetted 100 µm pore nylon filter (CMN-0105-D, Small Parts Inc., Logansport, IN, USA) and once through a pre-wetted 5 µm pore hydrophilic filter (CMN-0005-D, Small Parts Inc.) held in a 13 mm diameter filter holder (XX3001200, Millipore, MA, USA). The filtrate was centrifuged at 1000 × g for 10 min at 4 °C and the synaptoneurosome fraction was obtained and later resuspended in synaptoneurosome buffer containing 0.32 M sucrose and 1 mM NaHCO_3_. The protein concentration was estimated using the Pierce™ BCA Protein Assay Kit (Cat # 23227, Thermo Fisher Scientific). For western blotting, 15 μg of synaptoneurosomes was loaded onto 12% SDS-PAGE gels and run at 100 V for 90 min. The gels were transferred to methanol-activated PVDF membranes (GE Healthcare) at 115 V for 1 h 45 min and then blocked with 5% milk in TBST for 1 h at RT. The membranes were incubated with primary antibodies overnight at 4 °C, washed with TBST, and incubated with secondary antibodies for 1 h at RT. Primary antibodies used were: 1:1000; guinea pig monoclonal anti-GluD1 (#MSFR102510, Frontiers Institute), 1:1000; rabbit monoclonal anti-Cbln1 (#ab181379, Abcam), 1:1000; rabbit polyclonal anti-Neurexin1α (#ANR-031, Alomone Labs), 1:1000; rabbit monoclonal anti-HA (#C29F4, Cell Signaling Technology), 1:1000; rabbit monoclonal anti-β-Actin (#13E5, Cell Signaling Technology). After washing, the blots were treated with SuperSignal ™ West Pico PLUS substrate (Thermo Fisher) and developed using a ChemiDoc imaging system (Bio-Rad). The optical density was analyzed using ImageJ and normalized to that of β-actin.

### Drugs for in vivo experiments

D-serine (Acros Organics Cat # 227070250) and MK-801 (Sigma Aldrich Cat # M107) were dissolved in PBS to prepare stock solutions of 100 μg/μl and 10 μg/μl, respectively, and were administered at a dose of 30 μg and 1 μg, respectively, bilaterally in the CeA region in a volume of 0.3 μl. Cbln1 (R&D Systems Cat# 6934-CB) was dissolved in PBS to prepare a stock solution of 1 μg/μl and was administered at a dose of 250 ng bilaterally in the CeA.

### Stereotaxic surgery

Cannulation surgery was performed as described previously [21], with minor adjustments. The mice were anesthetized using isoflurane and positioned in a stereotaxic apparatus. Following skull exposure, a small aperture was created, and bilateral implantation of 26-gauge stainless steel guide cannulas into the CeA was performed. Stereotaxic coordinates for the procedure were set at: AP: −1.22 mm, ML: ± 2.75 mm, DV: −4.0 mm. Secure fixation of the guide cannulas onto the skull was achieved using stainless-steel screws and dental acrylic cement. Animals were assigned to the experimental groups after a post-surgery recovery period of 7–10 days. Verification of cannula placement was conducted after behavioral experiments by histological examination of fixed brain tissue using light microscopy.

### CFA inflammatory pain model

Inflammation was induced by intra-plantar injection of 10 μl of complete Freund’s adjuvant (CFA; Sigma, St. Louis, MO, USA) to the right hind paw. Animals injected with 10 μl saline in the left paw served as controls.

### Von frey filament test

Mechanical hypersensitivity of the mice was assessed by the Von Frey Filament test using an electronic Von Frey aesthesiometer (IITC systems). During habituation, the mice were placed in the testing chambers and conditioned to a rigid filament (from IITC systems). The test was performed by placing the mice on the perforated bottom of the test chambers with free access to their paws. The filaments were applied perpendicular to the plantar surface of the hind paw, and the paw withdrawal threshold was measured. The nocifensive response to filament application included licking, shaking, or brisk paw withdrawal. The force required for the nocifensive response was also noted. The average of three readings was taken at each time point.

### Statistical analysis

All experiments adhered to protocols ensuring uniform group sizes through randomization and blinded analysis. Statistical analyses were performed using the Prism 8.0 software (GraphPad Prism software, San Diego, CA, USA). The results were graphically represented as the mean value with standard error of the mean (SEM). Pairwise comparisons between two groups were conducted using the unpaired t-test, while multiple group comparisons were performed using analysis of variance (ANOVA) followed by Bonferroni’s post-hoc test for multiple comparisons.

## Results

### D-serine inhibits Cbln1 binding to GluD1 in an in vitro binding assay

**Fig. 1:**
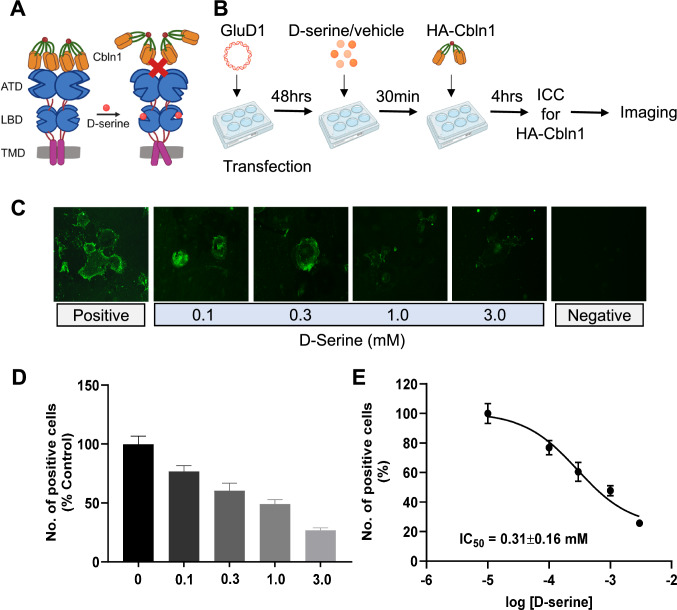
D-serine inhibits GluD1-Cbln1 interaction. **A** Schematic depicting the working hypothesis that D-serine inhibits the interaction between Cbln1 and GluD1. **B** Schematic workflow of the cell binding assay. Briefly, GluD1 overexpressing COS-7 cells were incubated with D-serine (0.1, 0.3, 1 and 3 mM) for 30 min, followed by incubation with HA tagged-rCbln1 for 4-h. Post-treatment cells were assayed for GluD1-Cbln1 binding using HA-tag immunocytochemistry. **C** Fluorescent images depicting the interaction between GluD1-Cbln1 (positive), while D-serine reduced HA labeling, suggesting that it impairs the interaction between GluD1 and Cbln1. **D** The percent inhibition of the GluD1-Cbln1 interaction was calculated as the number of HA-positive cells, normalized to the control, and plotted as a bar graph. D-serine reduced the number of positive cells in a concentration dependent manner (Experiment was repeated 3 independent times with two samples per group in each experiment). **E** Concentration–response curve of D-serine inhibition in HA-positive cells

**Fig. 2:**
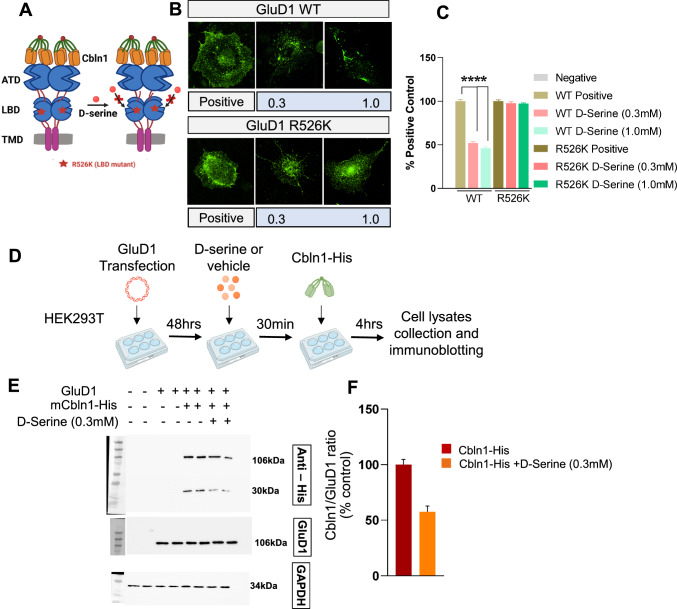
D-serine binding to the GluD1 ligand binding domain is necessary for preventing Cbln1 interaction with GluD1. **A** Schematic showing the hypothesis that D-serine has no effect on binding of Cbln1 with GluD1 R526K mutant. **B** Fluorescent images depicting the interaction between GluD1-Cbln1 (positive), while D-serine reduced HA labeling in the cells transfected with GluD1 WT construct, this was not observed with the mutant construct GluD1 R526K, suggesting that D-Serine binds to the ligand binding domain of GluD1 to produce this effect. **C** The percent inhibition of the GluD1-Cbln1 interaction was calculated as the number of HA-positive cells, normalized to the control, and plotted as a bar graph (Experiment was repeated 3 independent times with two samples per group in each experiment). **D** Schematic workflow of quantitative cell binding assay. **E** HEK293T cells were transfected with GluD1 and 48 h later treated with rCbln1-His for 4 h, with or without D-Serine (0.3 mM), followed by cell lysate preparation and immunoblotting to determine the inhibition of GluD1-Cbln1 binding by D-serine. Immunoblot showing the GluD1 (~106) and Cbln1 bands (~ 106 and 27) when probed with Anti-His antibody where the GluD1-Cbln1 binding was inhibited by D-Serine while no changes in GluD1 alone were observed. **F** Bar graph depicting the densitometric quantification of GluD1-Cbln1 (106 kDa) binding of Cbln1 only (positive control) and Cbln1 + D-Serine (n = 4). Data are shown as Mean ± SEM

Cbln1 is known to interact with the ATD of GluDs with high affinity to form a transsynaptic complex [22, 23]; however, the effect of D-serine on this interaction is not well understood. The interaction between GluDs and Cbln1 can be replicated in heterologous expression systems [2, 19]. Therefore, we assessed the effect of D-serine on the binding of rCbln1 to GluD1 in an in vitro cell-binding assay (Fig. 1A, 1B). A concentration–response curve was generated using 0.1, 0.3, 1- and 3-mM D-serine (Fig. 1C). We found that increasing concentrations of D-serine reduced the binding of rCbln1 to GluD1 (Fig. 1C and D). The IC_50_ for D-serine inhibition of the rCbln1-GluD1 interaction was 0.31 ± 0.16 mM. Thus D-serine inhibits rCbln1 binding to GluD1 in a concentration-dependent manner. To further confirm that binding of D-serine to the LBD of GluD1 is required for the observed reduction in rCbln1 interaction with GluD1, we compared wildtype and mutant GluD1R526K with impaired D-serine binding [8] in cell-binding assay. We found that in cell-binding assay the number of positive cells representing the binding of rCbln1 to wildtype GluD1 was reduced by D-serine at both 0.3 and 1 mM concentrations to ~ 50%. In contrast, no significant D-serine-induced change in the number of positive cells representing rCbln1 interaction with GluD1R526K was observed (Fig. 2B and C). These results demonstrate the requirement for binding of D-serine to GluD1 to impair the interaction between GluD1 and rCbln1.

As an alternative to testing GluD1-Cbln1 interaction using fluorescent assay we used an immunoblotting assay. Here we overexpressed GluD1 in HEK293 cells and applied rCbln1 with or without D-serine (300 µM) pre-treatment. After incubation periods as described in the methods, cells were washed and collected and thereafter probed in immunoblotting for GluD1 and Cbln1. We observed GluD1 bands in all wells transfected with GluD1 construct except the non-transfected sample confirming GluD1 expression. Cbln1 band probed by the His tag antibody was observed in samples where Cbln1 was applied either with or without pre-treatment with D-serine. Interestingly, a significant reduction in the Cbln1 binding was observed in samples from D-serine pre-treatment group suggesting a reduction in GluD1-Cbln1 interaction by D-serine (Fig. 2E and F).

### D-serine inhibits recombinant Cbln1-induced increase in excitatory neurotransmission in the central amygdala neurons

**Fig. 3:**
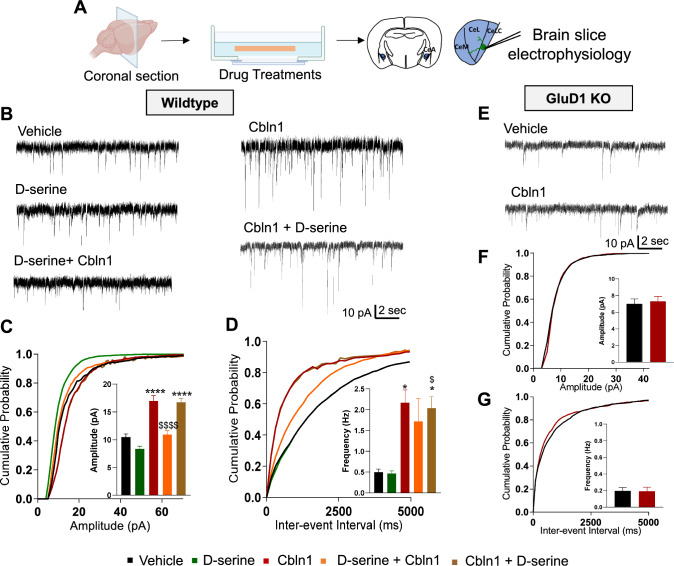
D-serine reduced the Cbln1 mediated increase in excitatory neurotransmission in the CeA. **A** Schematic depicting ex vivo brain slice experiments. Briefly, coronal sections were obtained using a vibratome and sections containing the CeA were incubated with different treatments, followed by electrophysiological recordings from neurons in the lateral capsular region of CeA (CeLC). **B** Representative traces of mEPSCs from neurons of WT animals exposed to different treatments; Vehicle (aCSF); D-serine (300 µM); rCbln1 (1 μg/mL) and both D-serine and rCbln1. Slices treated with both D-serine and rCbln1 were incubated with D-serine first for 15-min and followed by co-incubation with rCbln1 (D-serine + Cbln1) or rCbln1 first for 15-min and followed by co-incubation with D-serine (Cbln1 + D-serine). For all incubations, MK-801 was used to eliminate NMDAR-dependent effects.** C** Cumulative probability and bar graph of mEPSC amplitude. rCbln1 increased the amplitude of AMPA mEPSC in CeA neurons and this was prevented by pre-treatment with D-serine. However, D-serine had no effect when applied after Cbln1. (N = 8–9 neurons from 3 mice, One-way ANOVA; F (4, 38) = 30; Veh vs. Cbln1, ****p < 0.0001, Cbln1 vs D-serine + Cbln1, ^$$$$^p < 0.0001, Veh vs. Cbln1 + D-serine, ****p < 0.0001).** D** Cumulative probability graph for inter-event interval and bar graph for the frequency of mEPSC events. An increase in the frequency of mEPSCs was observed in rCbln1 treated CeA slices (One-way ANOVA; F(4, 38) = 7.648; Veh vs. Cbln1, *p = 0.0161). Pre-treatment of D-serine produced a modest but not significant attenuation and post treatment with D-serine had no effect on the rCbln1-induced increase in mEPSC frequency. **E** Representative traces of mEPSCs from GluD1 KO animals subjected to the rCbln1 treatment (1 μg/mL). **F** Cumulative probability and bar graph of mEPSC amplitude. Treatment with rCbln1 had no effect on the amplitude of the mEPSC in the GluD1 KO slices. **G** Cumulative probability and inter-event interval bar graph of mEPSC frequency. rCbln1 did not change mEPSC frequency in the GluD1 KO slices

Since D-serine prevents the binding of rCbln1 to GluD1, we next tested whether D-serine can block the downstream effects of rCbln1 in a native system. For these experiments, we developed an ex vivo brain slice model (Fig. 3A). Acute brain slices containing the CeA region were prepared. Slices were pre-treated with D-serine (300 µM) (or vehicle) followed by incubation with rCbln1 (or vehicle), as described in the Methods section. During the incubation of brain slices, MK-801 was included in the aCSF in all groups tested to eliminate the potential NMDA receptor-dependent effects of D-serine. After the incubation period, slices were transferred to the recording chamber, and mEPSC recordings were obtained from CeC neurons, which are known to be enriched in GluD1 [5]. Consistent with the known synaptogenic property of Cbln1, incubation with rCbln1 produced a robust increase in both the frequency and amplitude of mEPSCs in CeC neurons (Fig. 3B–D). D-serine application significantly reduced the rCbln1-induced increase in the mEPSC amplitude (Fig. 3C). In addition, pre-treatment with D-serine modestly, but not significantly, prevented the increase in the frequency of mEPSC produced by rCbln1 (Fig. 3D). We also tested the ability of D-serine in inhibiting rCbln1 effects on mEPSC post-rCbln1 application. D-serine did not prevent rCbln1-induced increase in mEPSC amplitude and frequency when applied post-rCbln1. It is likely that the synaptogenic effects of rCbln1 are not reversible once initiated. We also confirmed that the facilitatory effect of rCbln1 on excitatory neurotransmission were dependent on GluD1. Application of rCbln1 in GluD1 KO slices did not produce any change in excitatory neurotransmission in CeC neurons confirming that binding of rCbln1 to GluD1 is required for the effects on mEPSC (Fig. 3E–G). In preliminary studies we also explored whether application of rCbln1 in recording chamber increases excitatory neurotransmission. A significant increase in the amplitude of mEPSC was noted after application of rCbln1 in recording chamber suggesting rapid effect of rCbln1 (Supplementary Fig. 1). Further experiments are needed to standardize the rCbln1 application procedure to evaluate real-time effect of rCbln1.

### D-serine inhibits recombinant Cbln1-induced increase in GluD1 expression in the synaptic compartment in the central amygdala

**Fig. 4:**
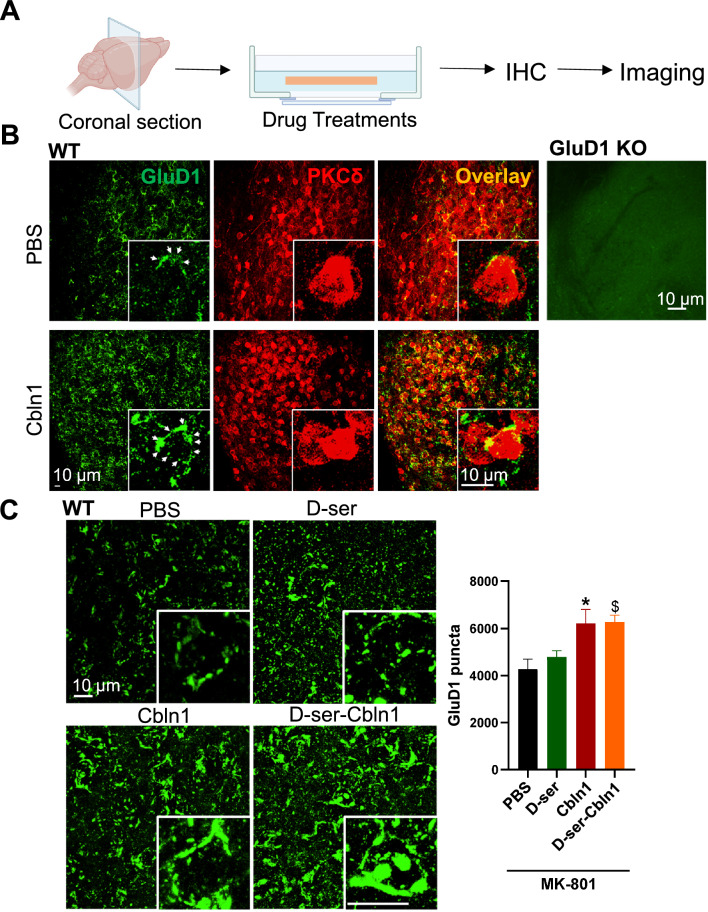
Cbln1 treatment increases GluD1 expression in PKC-δ neurons of the CeA. **A** Schematic illustrating the experimental design of ex vivo brain slice experiments. Coronal sections were obtained using a vibratome and sections containing the central amygdala (CeA) were incubated with various treatments, followed by immunohistochemical analysis. **B** Confocal images show an increase in GluD1 expression after rCbln1 treatment. GluD1 expression was preferentially localized in PKCδ- + ve neurons. The insets provide enlarged images of GluD1 and PKCδ expression or their overlay. The specificity of the GluD1 antibody has been validated in knockout animals. **C** Confocal images depicting GluD1 puncta in the CeA from the different treatment groups. D-serine alone did not affect GluD1 puncta or prevent the rCbln1-induced increase in GluD1 puncta. Statistical analysis using a one-way ANOVA revealed significant differences: F (3,21) = 6.335; MK-801-PBS vs. MK-801-Cbln1, **p = 0.0094; MK-801-D-serine-PBS vs. MK-801-D-serine-Cbln1, ^$^p = 0.0475

Due to its synaptogenic properties, it is conceivable that rCbln1 induces recruitment of GluD1, which then leads to the formation of synapses and an increase in excitatory neurotransmission. To test this hypothesis, we examined whether rCbln1 application affects GluD1 expression. We also explored whether D-serine could affect this phenomenon. Brain slice incubations were carried out in a manner similar to the electrophysiology experiment with MK-801 included in the incubation buffer for all groups, and after incubation, brain slices were mildly fixed and processed for IHC (Fig. 4A). We found that rCbln1 led to a robust increase in the expression of GluD1 in the CeA (Fig. 4B). The GluD1 elements upon rCbln1 application, as previously described, remained preferentially localized to PKCδ- + ve neurons (Fig. 4B). Interestingly, D-serine did not block the overall increase in the number of GluD1 puncta; however, in the presence of D-serine, GluD1 elements were denser, but this could not be quantified (Fig. 4C). Thus, D-serine is not able to block the initial synaptogenic effects of Cbln1, that is, recruitment of GluD1, but may partly reduce changes in synapse function, as evidenced by its ability to prevent an increase in mEPSC amplitude.

**Fig. 5:**
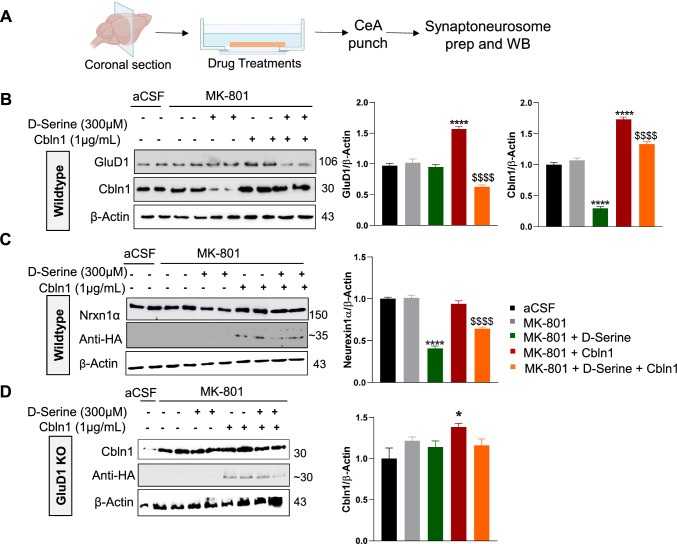
D-serine modulates synaptic expression of Cbln1 and Nrxn1α. **A** Schematic illustrating the experimental design of ex vivo brain slice experiments. Coronal sections containing the CeA were incubated with various treatments, followed by synaptoneurosome isolation and immunoblotting. **B** Immunoblot analysis and respective densitometry of GluD1, Cbln1. D-serine did not affect GluD1 expression (p = 0.99), but significantly reduced Cbln1 expression (p < 0.0001) compared to vehicle or MK-801. Conversely, rCbln1 significantly increased both GluD1 (p < 0.0001) and Cbln1 (p < 0.0001) expression compared with vehicle or MK-801. In slices treated with both D-serine and rCbln1, Cbln1 expression was elevated in these slices compared to the control, MK-801, or D-serine alone; however, it was significantly reduced compared to slices treated with rCbln1 alone. **C** Immunoblot analysis and densitometry of Nrxn1α and HA. rCbln1 did not affect Nrxn1α expression (p = 0.79), whereas D-serine significantly reduced Nrxn1α expression (p < 0.0001) compared to the vehicle- or MK-801-treated slices. This reduction was also observed in slices treated with both D-serine and rCbln1. The incorporation of rCbln1 into the CeA synapses was confirmed by immunoblotting for the HA tag, with bands observed only in the rCbln1-treated slices. aCSF vs. MK-801 + D-serine; ****p < 0.0001, MK-801 + Cbln1 vs. MK-801 + D-serine + Cbln1 ^$$$$^p < 0.0001. **D** Immunoblot analysis and respective densitometry of Cbln1 and HA in the central amygdala slices from GluD1 KO mice incubated with or without MK-801, D-serine and rCbln1-HA. Incubation with rCbln1 increased Cbln1 levels (*p = 0.0461). No significant difference in the Cbln1 was observed between rCbln1 only vs D-serine + rCbln1 incubated slices. Data are presented as the mean ± SEM; n = 10–12 slices per group, obtained from to 4–6 animals, with 3–4 slices pooled and counted as n = 1. Data was analyzed by one-way ANOVA and Bonferroni's post-hoc test

To gain a better understanding of the synaptic changes induced by D-serine and rCbln1 on the transsynaptic complex, we analyzed changes in GluD1, Cbln1 and Neurexin-1α (Nrxn1α) expression in the CeA synaptoneurosome fraction after incubation with various drug combinations (Fig. 5A). Interestingly, D-serine alone (in the presence of MK-801) produced a robust decrease in Cbln1 expression in synaptoneurosomes (Fig. 5B). However, there was no change in GluD1 expression by D-serine alone, which is consistent with the lack of effect of D-serine on AMPA mEPSC and GluD1 expression in IHC (Figs. 3C, 4C). Additionally, Nrxn1α, the presynaptic partner of the transsynaptic complex, was significantly reduced upon treatment with D-serine alone. Consistent with the IHC data (Fig. 4C), rCbln1 treatment resulted in a robust increase in GluD1 expression (Fig. 5B). In addition, rCbln1 was incorporated into the synaptic compartment, as evidenced by the presence of HA-tag labeling in the synaptoneurosome fraction after rCbln1 treatment (Fig. 5C). Interestingly, pre-treatment with D-serine blocked the rCbln1-induced increase in GluD1 in the synaptoneurosome fraction and partially blocked the increase in Cbln1. Together with the IHC data, these findings suggest that D-serine does not block the upregulation of GluD1 induced by rCbln1, but prevents its synaptic accumulation. We also conducted synaptoneurosome analysis in brain slices from GluD1 KO mice. Application of D-serine alone did not change the levels of rCbln1 in CeA synaptoneursomes from GluD1 KO suggesting that D-serine alone produces its effect on the expression of Cbln1 by binding to GluD1 and inducing downstream effects (Fig. 5D).

### Intra-CeA administration of D-serine inhibits nociceptive responses induced by recombinant Cbln1

**Fig. 6:**
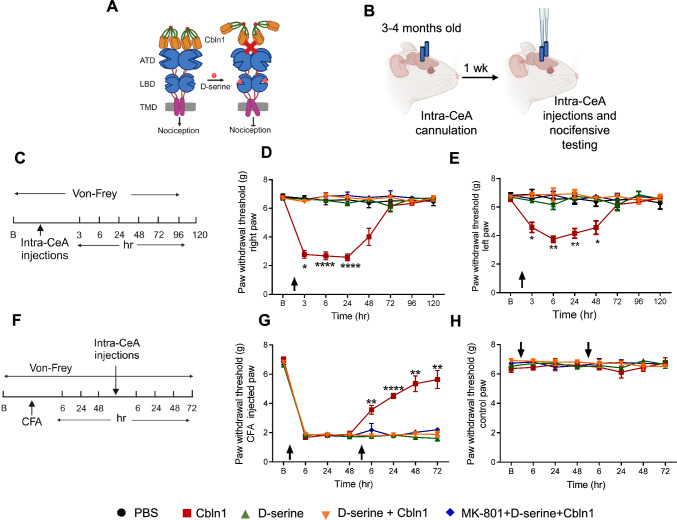
D-serine administration in the central amygdala counteracts the nociceptive effects of Cbln1. **A** Schematic depicting the working hypothesis that D-serine binding to GluD1 affects nociceptive signaling. **B** Schematic of the experimental design: C57BL/6 mice underwent cannulation surgery and were administered D-serine, rCbln1, and/or both into the CeA. The mechanical sensitivity was assessed using the von Frey filament test. **C** Timeline for assessment of mechanical hypersensitivity. The arrow represents the time of drug injection. **D** D-serine (30 µg/side) prevents the pro-nociceptive effect of rCbln1 (250 ng/side). Although D-serine itself did not affect the paw withdrawal threshold, it significantly abolished the hyperalgesic effect of rCbln1. Administration of MK-801 (1 μg/side) prior to D-serine and rCbln1 injection in mice did not change the effect of D-serine and/or Cbln1; N = 5 per group, two-way ANOVA, multiple comparisons, Bonferroni’s test, treatment F(14, 63) = 18.76, ****p < 0.0001 for 3 h, ****p < 0.0001 for 6 h, ****p < 0.0001 for 24 h, *p < 0.0261 for 48 h, *p = 0.0374 for 96 h. **E** A similar effect is observed in the left paw. Two-way ANOVA, multiple comparisons, ****p < 0.0001 for 3 h, ****p < 0.0001 for 6 h, ****p < 0.0001 for 24 h, *p < 0.0366 for 48 h, *p = 0.0374 for 96 h. **F** Timeline for the assessment of mechanical hypersensitivity in the inflammatory pain model: Baseline paw withdrawal threshold was recorded for 3 days, followed by intra-plantar injection of CFA. Intra-CeA administration of drugs was conducted 48 h after CFA injection. **G** D-serine antagonizes the analgesic effect of rCbln1 in an inflammatory pain model. D-serine alone did not increase the paw withdrawal threshold of the inflamed paw but significantly counteracted the effect of rCbln1 N = 5 per group, Two way ANOVA, multiple comparisons, Bonferroni’s test, treatment F(14, 84) = 28.66, **p = 0.0084 for 6 h, ****p < 0.0001 for 24 h, **p = 0.0056 for 48 h, **p = 0.0066 for 72 h** H** Paw-withdrawal threshold of the saline injected paw was unaffected

We have previously found that intra-CeA injection of rCbln1 in naïve animals produces mechanical hyperalgesia [5]. Importantly, this effect was absent in GluD1 knockout mice, suggesting that GluD1-Cbln1 transsynaptic signaling is essential for transmitting pain signals in naïve animals (Fig. 6A). Our in vitro results demonstrated that binding of rCbln1 to GluD1 was inhibited by D-serine. This finding was further supported by our ex vivo experiments, which showed that D-serine partly prevents the signaling induced by rCbln1. To examine the in vivo implications of this finding, we tested whether D-serine reduced the mechanical hypersensitivity induced by intra-CeA injections of rCbln1 (Fig. 6B). Naïve animals received intra-CeA injections of D-serine (30 μg in 0.3 µl) or vehicle. After a 30-min interval, 250 ng (in a volume of 0.3 µl) of rCbln1 (or vehicle) was injected into the CeA, and the animals were subjected to von Frey testing at various time points (Fig. 6C). Intra-CeA injection of rCbln1 produced a robust reduction in paw withdrawal threshold in naïve mice (Fig. 6D, E). Interestingly, mice that received pre-treatment of D-serine did not show mechanical hyperalgesia upon intra-CeA injection of rCbln1 (Fig. 6D, E). Thus, D-serine blocked the rCbln1-induced increase in mechanical hypersensitivity. Naïve animals injected with D-serine alone showed no change in paw withdrawal threshold (Fig. 6D, E). Moreover, injection of MK-801 into the CeA prior to D-serine and rCbln1 did not influence the outcome, suggesting that these effects were independent of NMDA receptor activation (Fig. 6D, E).

We have previously found that intra-CeA injection of rCbln1 produces an analgesic effect in an inflammatory pain model [5]. Therefore, we tested whether the effect of rCbln1 could be inhibited by D-serine. After baseline measurements of footpad mechanical sensitivity, CFA was injected into the right hind paw of naïve animals and mechanical sensitivity was tested at 6, 24, and 48 h post CFA injection (Fig. 6F). Thereafter D-serine followed by vehicle, vehicle followed by rCbln1 or D-serine followed by rCbln1 was injected bilaterally into the CeA on day 3 after CFA injection. Interestingly, we found that D-serine inhibited the analgesic effect of rCbln1 in an inflammatory pain model (Fig. 6G). Similar to the above experiment, the injection of MK-801 in the CeA, prior to D-serine + rCbln1, did not affect the results, suggesting that these effects were independent of NMDA receptor activation (Fig. 6G). The control paw (non-CFA-injected paw) did not show any change in the paw withdrawal threshold throughout the experiment (Fig. 6H).

## Discussion

Our study revealed that D-serine inhibits GluD1-Cbln1 interaction and signaling. Furthermore, the effect of D-serine on GluD1-Cbln1 signaling in ex vivo and in vivo studies is independent of NMDA receptor activation. In in vitro experiments, we found that D-serine produced a concentration-dependent inhibition of rCbln1 binding to GluD1 with an IC_50_ of  ~ 300 μM, which is consistent with previous reports of D-serine affinity for GluD1 [9]. In ex vivo brain slices, D-serine partly blocked the rCbln1-induced increase in excitatory neurotransmission and synaptic recruitment of GluD1. Moreover, in vivo studies demonstrated that the nociceptive action of rCbln1 in naïve animals was effectively counteracted by D-serine administration. Additionally, D-serine inhibited the analgesic effects of rCbln1 in an inflammatory pain model. Together, these data demonstrate that D-serine can inhibit rCbln1 from binding to GluD1 and initiating downstream signaling.

### Allosteric interaction between D-serine and Cbln1 binding

Based on our in vitro, ex vivo, and in vivo data, we propose that D-serine binds to the LBD of GluD1 and induces a conformational change that hinders the interaction between Cbln1 and GluD1, thus preventing the formation of a tripartite complex and thwarting GluD1-Cbln1 signaling. The GluD-Cbln1-Nrxn triad (~ 24 nm) occupies a tight synaptic cleft [22–24]. Cbln1 is a dimer of trimers, and each trimer interacts with one of the four ATDs of tetrameric GluD [2, 3, 22, 23]. Thus, the two hexameric molecules of Cbln1 bind to a single GluD. Upon binding of D-serine, changes similar to AMPA receptor desensitization occur, leading to breakdown of the ATD interface. In this scenario, the flexibility of hexameric Cbln1 may not be sufficient to bind two ATDs simultaneously, thereby reducing Cbln1 affinity for GluD1. Alternatively, it has been proposed that closure of the LBD of GluDs by D-serine contracts the GluD receptor towards the postsynaptic membrane [22]. This contraction may be responsible for the dissociation of the trans-synaptic complex. Given that Cbln1 binds to Nrxn with higher affinity (Kd ~ 47 nM) [3, 23] than to GluD1 (Kd ~ 100 nM), it is possible that Cbln1 dissociates from GluD1 but remains bound to Nrxn. The effects of D-serine alone on synaptoneurosome experiments (Fig. 4), as discussed below, are consistent with this hypothesis.

The majority of our data examined whether the rCbln1 interaction with GluD1 was inhibited by D-serine. The effect of D-serine alone in disrupting the existing trans-synaptic GluD1-Cbln1 interaction was not directly tested in a heterologous system. However, our results from brain slice experiments provide some clues. We observed an intriguing reduction in Cbln1 content in synaptoneurosomes after D-serine treatment alone. This implies that D-serine dissociates the transsynaptic interaction between GluD1 and Cbln1. As discussed above, since the affinity of Cbln1 is higher for Nrxn1α [3, 23], it is possible that upon dissociation from GluD1, the Cbln1-Nrxn1α complex may traffic to non-synaptic regions, thereby reducing the Cbln1 content in the synaptoneurosomes. This hypothesis is consistent with the complementary reduction in Nrxn1α expression in the synaptoneurosome fraction following D-serine treatment alone. However, it should be noted that D-serine alone did not produce any change in GluD1 content in synaptoneurosomes, GluD1 expression in IHC, or mEPSC characteristics. It is possible that a longer duration of incubation with D-serine may reveal functional changes in CeA synapses or GluD1 expression. Alternatively, it is possible that D-serine-induced changes in Cbln1 content in the synaptoneurosome are a transient pharmacological response. Also relevant is the observation that the intra-CeA injection of D-serine alone did not affect nociception. Thus, D-serine alone may not produce similar changes, such as a reduction in synaptic Cbln1, in vivo, as observed ex vivo. Alternatively, this could be attributed to the diffusion or metabolism of D-serine from the site of action, resulting in a lack of change in nociception 3 h post-D-serine injection. The synaptic concentrations of D-serine are typically in low micromolar range and D-amino acid oxidase (DAAO) enzyme can potentially reduce the concentration given intracranially rapidly [25]. Thus it is possible that D-serine present in CeA synapses gets degraded or uptaken which may restrict its effect in vivo. It will be interesting to evaluate whether physiological or pathological conditions involving a longer duration of exposure to elevated levels of D-serine may produce effects mediated by the redistribution of Cbln1.

### Robust effects of recombinant Cbln1 in ex vivo central amygdala slices

In electrophysiological studies, we found that rCbln1 application produced a dramatic increase in both the frequency and amplitude of mEPSC in CeA neurons. Given the increase in GluD1 expression upon rCbln1 application, we predicted that there would be an increase in synapse formation and not merely an increase in presynaptic transmitter release, which would account for the rCbln1-induced increase in the frequency of mEPSCs. The increase in the amplitude of mEPSCs suggests the incorporation of AMPA into the synapses. Although the direct effect of rCbln1 on AMPA receptor expression has not been examined in the CeA, it has previously been found that ablation of GluD1 or GluD2 leads to a contrasting increase in AMPA receptors [21, 26, 27]. Conversely, it is possible that overexpression or upregulation of GluD1 leads to reduced AMPA receptors. However, we found that short exposure to rCbln1 increased GluD1 expression and simultaneously increased the amplitude of AMPA receptor-mediated mEPSC, suggesting the recruitment of AMPA receptors. These effects of rCbln1 were observed in the presence of MK-801, suggesting that they were independent of NMDA receptor activation. Another observation in electrophysiological studies is the occurrence of large-amplitude currents by the application of rCbln1. These partly resemble the slow inward currents that are dependent on NMDA receptors and astrocytic function [28–30]. It is possible that axosomatic synapses with large postsynaptic appositions may be responsible for these large currents.

These plastic changes induced by rCbln1 were partly blocked by D-serine pre-treatment potentially by blockade of GluD1-Cbln1 interaction. Specifically, D-serine blocked the rCbln1-induced increase in amplitude of AMPA receptor mEPSC, but did not significantly inhibit mEPSC frequency. Furthermore, D-serine blocked the rCbln1-induced increase in GluD1 in the synaptoneurosome fraction but not the overall increase in GluD1 expression in IHC. Thus, D-serine may prevent synaptic changes induced by rCbln1, but may not affect non-synaptic changes or the establishment of new synapses by rCbln1.

### Implications of allosteric interaction between GluD1 LBD and Cbln1 binding on synaptic organization

One potential consequence of our results is that during early development, regions with high D-serine levels may limit the interaction between Cbln1 and GluD1, and therefore limit the range of GluD1-Cbln1 signaling in synaptogenesis. In the CeA, 39% of neurons express D-serine, and 68% express the serine racemase (SR) enzyme, suggesting a potential functional role for D-serine in this region [31]. Our study suggests that such a role may involve GluD1 in addition to NMDA receptors. Similarly, mechanisms that induce the release of D-serine from glia or neurons may compete with the binding of Cbln1, released in an activity-dependent manner, to GluD1 and the consequent synaptogenic function. This may allow other synaptogenic factors to mediate the synapse formation. It will also be interesting to test the effect of GABA, which was recently shown to bind to the GluD1 LBD on rCbln1 binding to GluD1 [9].

## Conclusions, limitations and future perspectives

Our study has certain limitations. While the in vitro cell-binding assay sheds light on the interaction between Cbln1 and GluD1 in the presence of D-serine, it does not fully capture the complexity of in vivo synaptic conditions. Although ex vivo and in vivo studies substantially mitigated this concern, further research is needed to ascertain whether the concentrations of agents used are physiologically relevant and if similar effects are evident at lower levels that are biologically pertinent. Additionally, our ex vivo studies focused on the short-term effects of D-serine and rCbln1 on excitatory neurotransmission and synaptic changes. Long-term investigations are necessary to determine whether these effects are sustained over time and to explore their potential impact on chronic pain conditions and synaptic plasticity. In conclusion, our results demonstrate that D-serine inhibits the effects of rCbln1, suggesting a potential for modulating pain pathways and other physiological processes regulated by GluD1-Cbln1 interactions.

## Supplementary Information

Below is the link to the electronic supplementary material.

Supplementary file1 (PDF 637 KB)

## Data Availability

The data supporting the findings of this study are available from the corresponding author upon reasonable request.

## Abbreviations

CeA: Central amygdala
GluD: Glutamate delta
Cbln: Cerebellin
ATD: Amino Terminal Domain
LBD: Ligand Binding Domain
CTD: C-terminal domain
iGluRs: Ionotropic glutamate receptors
NMDA: N-methyl-D-aspartate
AMPA: α-Amino-3-hydroxy-5-methyl-4-isoxazolepropionate
