# Supplementary figures and images for: D-Serine disrupts Cbln1 and GluD1 interaction and affects Cbln1-dependent synaptic effects and nocifensive responses in the central amygdala
Source: Cell Mol Life Sci. 2025 Jan 31;82(1):67. doi: 10.1007/s00018-024-05554-z (PMC11785871; doi:10.1007/s00018-024-05554-z)

**A**

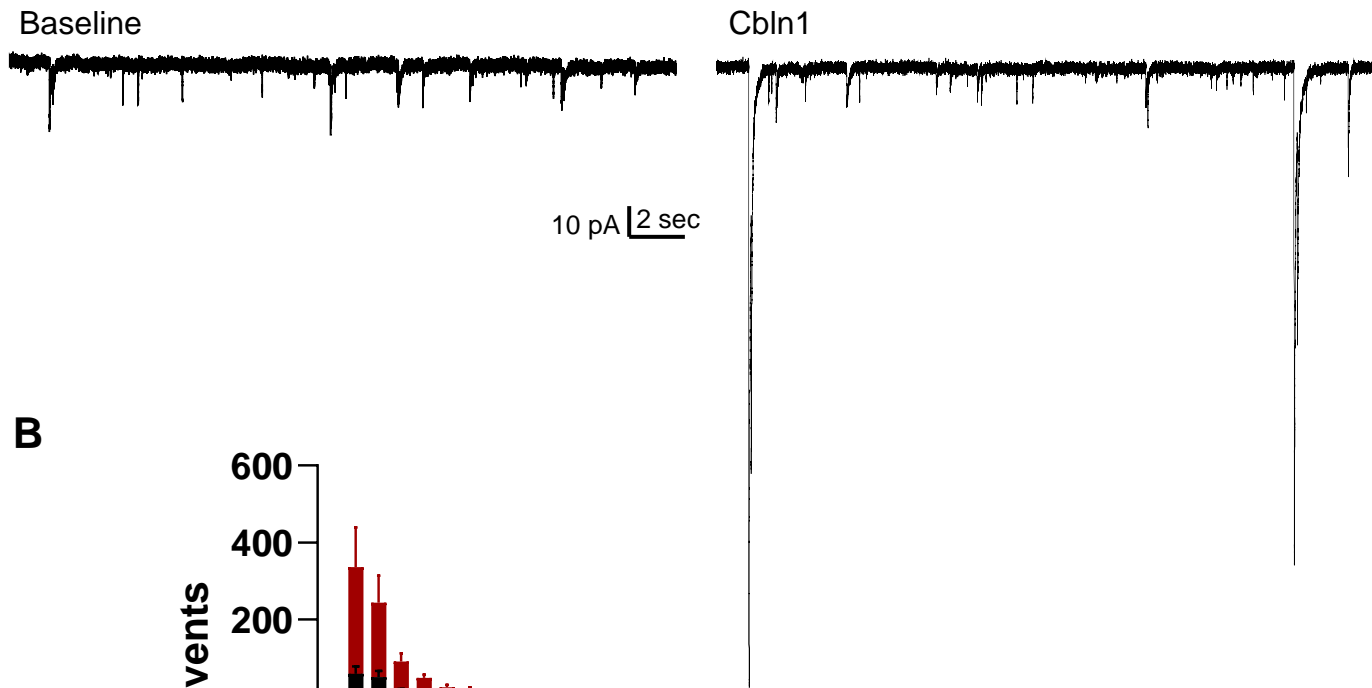

**B**

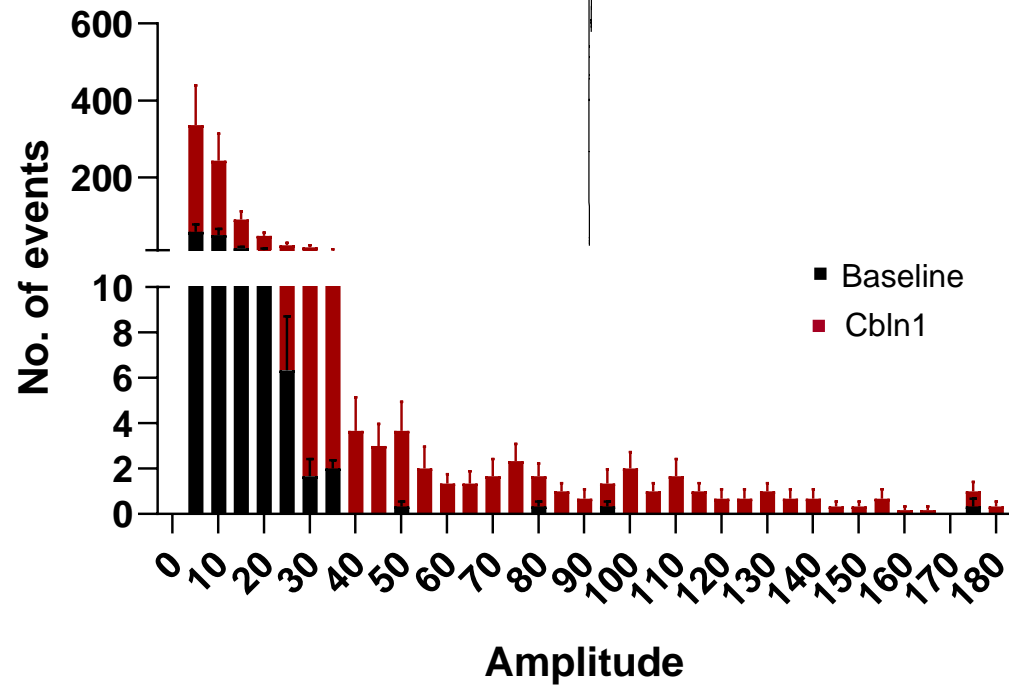

Supplement: Supplementary file 1 — Supplementary file1 (PDF 637 KB) [file 18_2024_5554_MOESM1_ESM.pdf]
